# Perspectives on antenatal education: a commentary

**DOI:** 10.55975/QDCU6150

**Published:** 2025-09-01

**Authors:** Bookless Rebekah, Charlton Naomi, Smith Joanne, Doherty Alison, Harrison Joanna, Hill James Edward

**Affiliations:** 1 Liverpool Womens NHS Foundation Trust; 2 Alder Hey Children’s Hospital Trust; 3 Liverpool City Council; 4 University of Central Lancashire; 5 University of Central Lancashire

## Abstract

This commentary critically appraises a systematic review that assessed the impact of antenatal education on pregnancy (physical and mental health) outcomes, and on physical health outcomes for infants in the perinatal period. The commentary considers the review’s findings alongside other existing related research, which may provide insights for the further development of antenatal education, and the latent potential to improve outcomes for prospective parents and infants.

## Introduction

There are approximately 600,000 births in NHS hospitals each year (1). Giving birth is one of the most stressful events for prospective parents (2). Antenatal education provides a key point of contact with prospective parents and offers them information about pregnancy, birth, and the postnatal period (3). It has the potential to improve their outcomes and experiences through education and skill development (4; 5). Antenatal programmes have existed for over 50 years in the UK (6). Today, prospective parents can access a range of antenatal education delivery modes and information sources (7; 8). However, consensus around antenatal education remains elusive (9), outcomes are largely unknown (10), and standardisation is needed to ensure quality of provision (11; 12). A systematic review by Hong et al., 2021, aimed to gather available evidence to explore the impact of antenatal education programmes on pregnancy (physical and mental health) outcomes, and infant physical health outcomes (13).

### Methods of the review by Hong et al., 2021

Hong et al., (2021) performed a systematic review and meta-analysis. Five bibliographic databases were searched from inception to November 2018 following PICO criteria: **P**opulation (pregnant women); **I**ntervention (antenatal education), **C**omparison (not specified), and **O**utcome (maternal and foetal outcomes including physical or mental health components). Studies eligible for inclusion included retrospective, population-based, cross-sectional, cohorts and case-controlled studies, observational, Randomised Controlled Trials (RCTs) or non-RCTs. Three pairs of reviewers independently screened studies for eligibility. Each pair of reviewers independently risk assessed studies using recognised quality assessment tools and extracted data from the included studies. Any disagreements during these review stages were resolved by third-party consensus. A meta-analysis, sub-group analyses, and a sensitivity analysis were performed. The statistical heterogeneity of the studies was assessed using Higgins’ I^2^ statistic, and Cochrane’s *Q*-test. Subgroup analyses were performed to investigate methodological and clinical heterogeneity. Publication bias was assessed using funnel plots and ‘trim and fill’ analysis.

### Findings of the review by Hong et al. 2021

Twenty-three studies were included in the review (12 RCTs, two Non-RCTs, eight cohort studies, and one case control study). The studies’ antenatal education programmes were wide-ranging but mainly included structured childbirth classes to educate parents. The timing of the interventions varied. Outcomes reported by the studies included pregnancy (physical and mental health) outcomes, and infant physical health. Six observational studies included in the review were assessed as being of low risk of bias. The remaining three observational studies were assessed as being of high risk of bias, low quality or unclear. Of the fourteen controlled trials – nine were assessed as high quality and five were assessed as being of low quality.

Twenty studies were included in a meta-analysis (14 controlled trials, five cohort studies, and one case control study). The three remaining studies were descriptive-only studies with no comparisons.

### Effects of antenatal education on physical pregnancy outcomes

The meta-analyses reported on physical outcomes including use of analgesics and of epidural anaesthesia; caesarean birth rate; and Apgar score or low birth rate.

#### Use of analgesics and epidural anaesthesia

The use of analgesics during labour was reported in four controlled trials and three observational studies, using a fixed-effect model and a non-significant pooled relative risk (RR) (0.893; 95% Confidence Interval (CI), 0.80-1.07). Analgesic use was not reduced in the antenatal education groups, but there was a statistically significant lower use of epidural anaesthesia in the antenatal education groups (Heterogeneity: *I^2^* = 76%; RR, 0.92; 95% CI, 0.88-0.97).

#### Effect of antenatal education during pregnancy on caesarean birth-rate

The effect of antenatal education during pregnancy on the caesarean birth rate was significantly low (Heterogeneity: *I^2^* = 36%; RR, 0.88; 95% CI, 0.80-0.96) with a fixed effect model.

#### Sub-group analyses

Sub-group analyses found social and / or emotional antenatal support programmes were effective at reducing the caesarean birth rate (Heterogeneity: *I^2^*=36%; RR, 0.86, 95% CI, 0.75-0.98) and beneficial for reducing epidural anaesthesia during labour (Heterogeneity: *I^2^*=76%; RR, 0.69, 95% CI, 0.51– 0.92), whereas practical educational programmes were not.

#### Infant Physical Health

The effect of antenatal education on infant physical outcomes was reported using the Apgar score or birth weight. The absence of heterogeneity in the analyses implied that antenatal education had no effect on the Apgar score. No differences were found in birth weight outcomes.

### Effects of antenatal education on mental health

Mental health reported outcomes included anxiety, stress, depression, self-efficacy, and satisfaction with the birthing experience. No meta-analyses were conducted for these outcomes - outcomes were assessed through self-completed questionnaires. Satisfaction with the birthing experience was reported as a positive outcome for antenatal education groups in four studies. Significant decreases in stress levels were identified in the antenatal groups of three of the five studies that evaluated stress. Two of the three studies which measured self-efficacy reported significant differences in the antenatal groups compared to controls. No differences were found for anxiety or depression.

### Commentary

Using the Joanna Briggs Institute of Critical Appraisal tool for systematic reviews (14) seven of the eleven criteria were judged to be satisfactory for this review. The authors did not publish a protocol in advance of their review in accordance with recommended guidance for reviews (15). Protocols specify, in advance, the scope and methods to be used by the systematic review, and their publication promotes transparency, reduces potential review authors’ biases and duplication of research by others, and assists with issues of reproducibility and reliability (16). The review’s searches were conducted in 2018 and not updated before publication. Searches for systematic reviews should be conducted within 12 months before publication (16; 17). The review excluded studies not published in the English Language without justifying reasons for doing so (despite including a Korean search term and searching a Korean database). The low number of identified publications (129 publications) suggests that the review’s search strategy is unlikely to be comprehensive as evidence is likely to have been missed. It is therefore doubtful that an accurate summary of the available evidence is provided. Furthermore, recommendations for policy and practice were not supported by the data and no specific directives for new research were provided.

### Main findings in context to practice

The review by Hong et al., 2021 was, to the best of our knowledge, the first review to investigate antenatal education programmes on both pregnancy (physical and mental health) outcomes, together with physical outcomes for infants. The review found statistically significant links between antenatal education and certain outcomes including epidural use, mode of birth, self-efficacy, and stress. However, the review’s limitations make these findings inconclusive, and it is not possible to make recommendations for practice based solely on this review.

An earlier systematic review which explored the effects of antenatal education programmes on reducing fear of childbirth outcomes found limited evidence of their effectiveness and concluded that more appropriately randomised trials are needed (18). A recent scoping review found evidence to suggest that providing antenatal education and routine care compared to routine care (only) may decrease the fear of childbirth, postpartum depression, and pain intensity during labour, but these review’s authors similarly found that the identified studies lacked sufficient quality to make definitive conclusions (19).

A criticism of antenatal education is that it is based on what information healthcare professionals feel should be imparted rather than on the information needs of participants (20). A study which explored participants’ experiences of engaging in online antenatal education found that they want trustworthy and accurate information delivered within a framework underpinned by adult-learning principles (7). A recent integrative review found individuals at risk of inequities in online antenatal education provision, as well as digital health literacy needs for healthcare professionals (21).

Some advances in the field of antenatal education are being made. For example, a UK project has informed the development of a service delivery model for online antenatal education (11). In Australia, antenatal education guidelines which align with adult-learning principles have been developed (22). However, there is a lack of evidence-based recommendations for antenatal education programmes within the guidelines (23).

### Recommendations for future research

Robust high-quality studies are needed to explore antenatal education programmes, and their effects on specificized outcomes. Minority and vulnerable groups are under-represented in this research field (8) and may be less likely to access antenatal education (24). Research is therefore needed to understand how to address issues faced by such groups (21).

The review by Hong et al., 2021 found some evidence to suggest that social or emotional support programme types are effective at reducing the caesarean birth rate and the use of epidural anaesthesia during labour. Future research may usefully explore these types of programmes.

A realist review may help to highlight the human and external factors which influence effectiveness and outline why there are differences in practice. Qualitative research can usefully explore the views and experiences of recipients and providers of antenatal education (25). Implementation science can guide the design and implementation of antenatal education programmes (11).

Research in this field is ongoing: a search conducted by this commentary’s authors of clinical trials registries (ISRCTN Registry; EU Clinical Trials Register - Update) and of the PROSPERO register of review protocols PROSPERO (york.ac.uk) identified over 30 review protocols and two clinical trials related to antenatal education. Topics being investigated include, for example: the effects of antenatal education on partners; the perspectives of healthcare professionals involved in facilitating antenatal education; and internet-based antenatal education. This research (once completed and published) may provide research evidence to inform future antenatal education developments and outcomes.

## Conclusions

▪ Antenatal education may improve pregnancy outcomes for stress and child-birth self-efficacy, may lower the caesarian birth rate, and decrease the use of epidural anesthesia.
▪ Currently, studies investigating the effects of antenatal education are, however, difficult to interpret due to their methodological and clinical heterogeneity.
▪ Robust research is needed to elucidate the effects of antenatal education, to ensure quality provision, and standardization of antenatal education.

### Practice challenges

1. How should the effectiveness of antenatal education be measured?
2. How should antenatal education be developed?
3. Is it possible to develop standardised guidance on antenatal education given existing differences in practice?
